# Cervical cancer screening management practices and prevention in uMsunduzi Local Municipality primary care clinics

**DOI:** 10.4102/hsag.v27i0.1934

**Published:** 2022-11-09

**Authors:** Bhekuyise L. Mncube, Sipho W. Mkhize

**Affiliations:** 1 School of Nursing and Public Health, College of Health Sciences, University of KwaZulu-Natal, Durban, South Africa

**Keywords:** cervical cancer, cervical cancer screening, knowledge of cervical cancer, management practice, nurses

## Abstract

**Background:**

Although cervical cancer is preventable, it remains the most feared and second most common cancer in women worldwide, as well as the leading cause of cancer deaths in many low- and middle-income countries, including South Africa. Numerous studies conducted globally, in Africa and in South Africa revealed a knowledge gap about cervical cancer and its preventive strategies, including cervical cancer screening, among nurses and the general population.

**Aim:**

The purpose was to investigate and describe nurses’ knowledge and management practices regarding cervical cancer screening in uMsunduzi Local Municipality, KwaZulu-Natal.

**Methods:**

The study was directed by a positivism paradigm. A quantitative research approach and nonexperimental descriptive design was used in this study. Probability random sampling was used, and a self-administered questionnaire was used to collect data. Eighty-three professional nurses participated in the study.

**Results:**

The study discovered that professional nurses working in selected clinics in uMsunduzi Local Municipality, KwaZulu-Natal, had a high level of knowledge about cervical cancer and its screening. Nonetheless, despite the high level of knowledge demonstrated, the level of practice remained low.

**Conclusion:**

Cervical cancer morbidity and mortality have long been a major health concern in South Africa’s general population. The study found that PNs are knowledgeable about cervical cancer screening; conversely, practice was low. This paper includes recommendations for future research, nursing practice, nursing education and the Department of Health.

**Contribution:**

This study contributed vastly to the body of knowledge in managing cervical cancer screening practices, particularly in health promotion and prevention of diseases at primary care level.

## Introduction

Cervical cancer is preventable, but it is still the most feared and second-most common cancer in women worldwide, as well as the leading cause of cancer deaths in many low- and middle-income countries, including South Africa (Khoza et al. 2016:1:3). According to Khoza et al. (2016:3) and Mignote et al. (2015:1), cervical cancer is the second-most common cause of morbidity and mortality among gynaecological cancers worldwide. Cervical cancer is defined as the growth of abnormal cells in the cervix lining (Cancer Council South Africa 2017:3).

According to the World Health Organization (2015, 2022; Storr 2015), there are approximately 604 000 cases of cervical cancer each year, with 342 000 deaths worldwide in 2020. In Africa, it was estimated that approximately 80 000 women were diagnosed with cervical cancer each year, with over 50 000 women dying as a result of the disease (Zengwe 2016:14). According to Zengwe (2016:15), nearly 70% of women diagnosed with cervical cancer die in Africa. In most African regions, the estimated standardised incidence rate was approximately 31.5 cases per 100 000 women, compared to 6.3 cases per 100 000 women in the majority of European and American regions (Olorunfemi 2017:1).

Cervical cancer detection at an early stage is considered life-saving because it reduces cervical cancer mortality by 60% – 90% (Anyebe et al. 2014:34). Nurses working in various public health facilities, particularly primary healthcare (PHC) facilities, are trailblazers in the management of cervical screening programs and practices (Anyebe et al. 2014:6). Anyebe et al. (2014) believed that in the South African context, a study was needed to reflect on nurses’ knowledge and management practice of cervical cancer screening (CCS).

Bogers et al. (2017:4) stated persuasively that nurses working in public healthcare institutions played a critical role in managing and executing CCS programs, performing cervical screenings (Pap smear), health welfare promotion and cervical cancer (human papilloma virus) vaccinations. Investigating CCS knowledge and management practices among nurses working in public PHC facilities was critical to determining their potential, impact and effect in the delivery of cervical screening measures, as well as combating morbidity and mortality associated with cervical cancer (Goyal et al. 2013:253).

The research was guided by a logic framework approach (LFA) adapted from Finley (2015). According to the framework, the outcomes of practice are directly affected by available assets and recommended processes. Nurses’ knowledge on CCS has enormous prospective impact on the practices’ outcomes. Thus, this framework was of paramount prominence in investigating nurses’ knowledge and management practice of CCS.

Investigating nurses’ knowledge and management of CCS practices in primary care clinics was considered crucial to identify knowledge deficit and to describe specific measures to improve management practices of CCS among nurses, which are the aim and objectives of this study.

### Purpose of the study

The purpose of this research was to investigate professional nurses’ knowledge and management practices regarding CCS and prevention in the uMsunduzi Local Municipality of KwaZulu-Natal.

### Objectives of the study

The study’s objectives were to assess knowledge regarding management practices of CCS and prevention among nurses, as well as to describe knowledge regarding management practices and prevention of CCS among nurses.

## Methodology

### The paradigm of research

The study’s goal was to collect information on what was known and done about CCS knowledge and management practices. The study was guided by the positivism paradigm to the answers sought through the application of the principle of reductionism by breaking human function down into knowledge and practice (Artino, Konge & Park 2020:691).

### Design and methodology of research

A quantitative approach and nonexperimental descriptive design were used by the researcher. The quantitative approach was used because it is a recommended method for investigating answers to research questions in order to produce accurate findings through measurement of research variables such as CCS knowledge and management practice (Rahman 2017:105). A nonexperimental descriptive design provided guiding principles to study the research problem and uphold the validity of the findings (Brink, Van der Walt & Van Rensburg 2015, 2018).

### Setting and population

The research was carried out at uMsunduzi Local Municipality, which has urban, semi-urban and rural PHC clinics in the uMgungundlovu district of KwaZulu-Natal province. The study’s population consisted of 100 professional nurses.

### Method of sampling

Regarding probability in this study, simple random sampling was used, which gave each respondent an equal and independent chance of being drawn. An accessible population was identified and listed, thus limiting incidents of bias in selection and subsequently promoting the reliability and validity of results.

#### Size of the sample

The Raosoft sample size calculator was used to calculate the population size under consideration. Raosoft’s sample size calculator accepts a 5% margin of error and a 95% confidence level (Omair 2014). A total population of 100 was entered into the Raosoft calculator, yielding a sample size of 80, which was more likely to represent the population of the study. A total of 83 people took part in this study, ensuring data redundancy.

#### Recruitment of respondents for the study

Recruitment of respondents was done by the researcher solely, and it took 10–20 min to recruit each potential respondent. Professional nurses were approached one-on-one in their PHC facilities to explain the study’s purpose, significance, objectives and how they would benefit from the study, requesting their participation. A letter of information was given for detailed information. Nurses willing to participate were sampled randomly using a table of random numbers. Those who were randomly selected and interested in participating were given an informed consent form to acknowledge their participation as voluntary by providing their signature after reading the consent form.

### Method of data collection

The respondents’ data were gathered using a self-report questionnaire that was adapted from the study by Kieti (2016:1). The questionnaire’s reliability and validity were tested through a pilot study that consisted of 10 respondents recruited from two PHC facilities within uMsunduzi Local Municipality. The pilot study’s findings were found to be reasonably accurate, consistent and sound; consequently, the questionnaire was considered reliable and valid. No changes were made to the adapted questionnaire. The findings of the pilot study are not included in the main study.

The questionnaire was divided into three sections:

- Section 1 focuses on the demographic data of the nurses and has six items as variables (gender, age, marital status, highest level of professional education, job title and working experience).
- Section 2 focuses on knowledge of cervical screening practice by responding to yes-or-no questions; five variables were tested (overall knowledge of CCS, sources of information about CCS practice, knowledge on methods for cervical cancer detection, frequency of Pap smears for HIV-negative women and frequency of Pap smears for HIV-positive women).
- Section 3 has 13 item statements as variables focuses on CCS management practices using a Likert scale.

### Data analysis

A total of 85 questionnaires were distributed to PNs, and 83 were appropriately completed, representing a 98% return rate, and this formed the basis for analysis. Each questionnaire in this study was assigned a numerical code. The data were presented and analysed using Microsoft Excel 2013 and the Statistical Package for Social Science (SPSS) software version 25, as well as the assistance of a co-researcher. No discrepancies between data were noted. The analysed data are displayed in the form of tables, graphs and charts. The full description can be found in the results and discussion sections.

#### Informed consent

The informed consent form was handed to willing potential respondents to give consent by signing the form at the end to confirm that they understood and were willing to conform to the purpose and procedure of the study.

#### Anonymity and confidentiality

Respondents were requested not to provide their names or positive identification on the information obtained; codes would be used.

#### Privacy

Data were protected from unauthorised persons, only the researcher and researcher’s supervisor having access to it; no names were linked to the data.

#### Autonomy

Respondents had the right to withdraw at any time if they so desired, without repercussion or penalty.

#### Beneficence

PNs benefitted from acumen on the implications of knowledge and management practices regarding CCS.

#### Justice

Respondents were selected randomly.

### Ethical considerations

All necessary and required permissions and approvals were obtained from KwaZulu-Natal Department of Health Research Committee (10 September 2019) and the University of KwaZulu-Natal, Human and Social Sciences Research Ethics Committee (02 October 2019) (reference number: HSS/O407/019M).

## Results

The findings were presented in the form of tables and graphs, and they are discussed.

### Section 1: Demographic factors

The study findings revealed that the most common gender of respondents in the clinics was female, epitomising the idea that the nursing profession is still dominated by the female gender. Most respondents were over the age of 40, and more than half were married. The majority of respondents had only a diploma as the highest level of professional education, and a greater number of them had working experience of 16 years and above. Table 1 presents frequencies and percentage data on gender, age and marital status, as well as highest level of professional education, job title and working experience of respondents.

**TABLE 1:** Descriptive summary of respondents’ demographic factors.

Items	Frequency *N* = 83	Percentage
**Gender**		
Male	11	13
Female	72	87
**Age**		
25–30	7	8
31–34	9	11
35–39	15	18
40–44	17	20
45–50	8	10
51–54	13	16
55–60	13	16
61 and above	1	1
**Marital status**		
Married	42	51
Single	39	41
Divorced	3	4
Widow or widower	3	4
**Highest level of professional education**		
Diploma	65	78
First degree	18	22
**Job title**		
Professional nurse	50	60
Senior professional nurse	26	31
Nurse manager	7	9
**Working experience**		
Less than 2 years	6	7
3–5 years	12	15
5–10 years	15	18
11–15 years	19	23
16 years and above	31	37

### Section 2: Knowledge of cervical screening practice

#### Sources of information about cervical cancer screening practice

**FIGURE 1:**
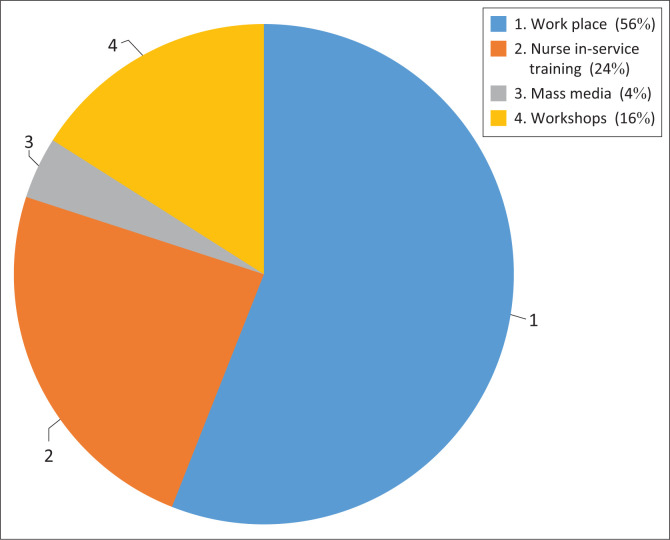
Sources of information about cervical cancer screening practice.

More than half of the respondents had obtained knowledge on CCS at their workplace. Almost a quarter of them attended nurse in-service training to acquire knowledge on CCS. Workshops were scored 16% by respondents, while a few attained knowledge through mass media platforms. Figure 1 presents respondents’ responses.

#### Knowledge about cervical cancer screening

Overall knowledge about CCS-related factors included cervical cancer risk factors, symptoms, prevention, screening methods, benefits of screening, recommended screening frequencies and available treatment. Table 2 indicates that basically half of the respondents had good knowledge of CCS and prevention, while some had very good knowledge on the phenomenon; only a few had poor to very poor knowledge. These findings expressed a high level of awareness and knowledge among respondents in clinics within uMsunduzi Municipality.

**TABLE 2:** Knowledge about cervical cancer screening.

Knowledge about cervical cancer screening	Frequency *N* = 83	Percentage
Very poor	2	3
Poor	4	5
Good	41	49
Very good	36	43

**Total**	**83**	**100**

#### Knowledge on detection of cervical cancer using Pap smear

The majority of respondents knew that a Pap smear was used to detect early cervical lesions, and they were aware that cervical cancer is curable when detected early; see Table 3.

**TABLE 3:** Early detection of cervical cancer using Pap smear and curability of cervical cancer when detected early.

Knowledge on early detection and curability of cervical cancer	Responses	Frequency *N* = 83	Percentage
Detection of cervical cancer using Pap smear	Yes	82	99
	No	1	1
	Not sure	0	0
Cervical cancer curable when detected early	Yes	79	94
	No	2	2
	Not sure	3	4

#### Knowledge on frequency of Pap smears for HIV-negative women

**FIGURE 2:**
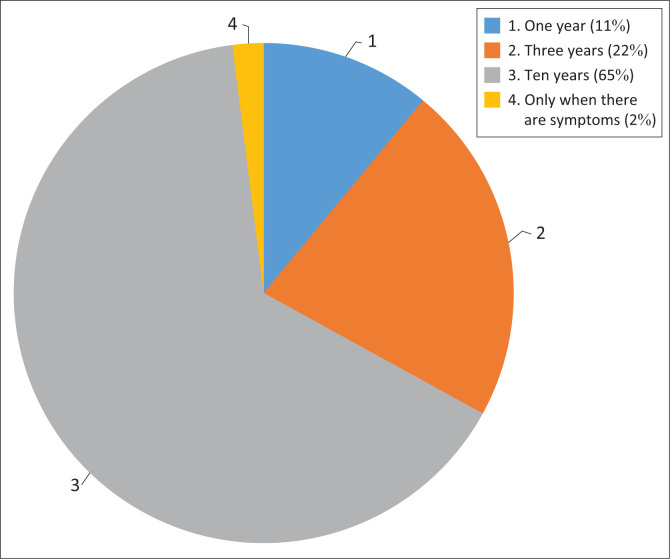
Responses on frequency of Pap smears for HIV-negative women.

The respondents were required to indicate the time intervals (in years) at which Pap smear tests should be performed for HIV-negative women. Figure 2 illustrates that the majority of respondents knew that in South Africa, a Pap smear is done at a 10-year interval for eligible women. Conversely, 22% of respondents responded with ‘3-year interval’, and some respondents (11%) selected ‘1-year interval’.

#### Knowledge on frequency of Pap smears for HIV-positive women

**FIGURE 3:**
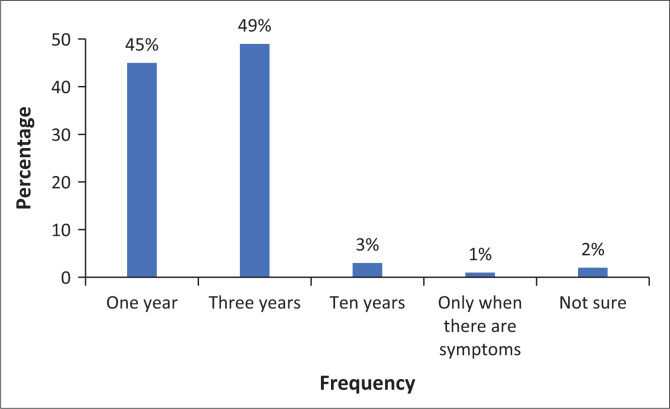
Responses on frequency of Pap smears for HIV-positive women.

The respondents were required to indicate the time intervals (in years) at which Pap smear tests should be performed for HIV-positive women. Figure 3 illustrated there was only a slightly difference between answers: ‘1 year’ scored 45%, and ‘3 years’ had 49%. These findings will be explained fully in the discussion.

### Section 3: Management practices of cervical cancer screening

Using a Likert scale, respondents were required to indicate how CCS practices were implemented in their respective PHC facilities (Table 4). The remarkable highlights of these findings were that the majority of PNs (82%) proclaimed they were adequately knowledgeable about CCS practices, and others stated that the required resources for CCS practice were often available. In terms of the actual performance of Pap smear tests, the majority of respondents revealed that there were hindrances to the actual practice of Pap smears, and some respondents preferred to be neutral. The results also revealed that a large number of respondents in the clinics participated in the promotion of CCS in sexually active women, as well as being involved in cervical cancer awareness campaigns facilitated by their PHC facilities. The findings further showed that most respondents in the clinics felt capable in implementing CCS practices; however, they were not confident enough to say that they were experts in CCS practice. An extent of 22% of PNs indicated good compliance in implementation of CCS practice, whereas a majority (32%) reported a lack of practice; however, there were those who were neutral (24%). Lastly, numerous nurses responded that CCS practices are often evaluated and monitored for standard compliance; conversely, the productivity of evaluation and monitoring seems indistinct.

**TABLE 4:** A summary of respondents’ responses regarding management of cervical cancer screening practices using Likert scale (Response = 100%).

Statements	Strongly agree (5)	Agree (4)	Neutral (3)	Disagree (2)	Strongly disagree (1)
4.3.1. Professional nurses are always sufficient and available to carry out cervical cancer screening practices	28%	37%	8%	16%	11%
4.3.2. There are adequate and available resources to carry out cervical cancer screenings	41%	35%	4%	13%	7%
4.3.3. I utilise available cervical screening materials cost-effectively	36%	48%	13%	1%	2%
4.3.4. I carry out cervical cancer screening practice as part of my day-to-day duties	39%	33%	14%	12%	2%
4.3.5. I perform two or more Pap smears per day	22%	22%	24%	25%	7%
4.3.6. Women who have been screened for cervical cancer always come back for results	17%	31%	22%	25%	5%
4.3.7. I adhere to referral protocol for smear-positive patients	48%	38%	12%	1%	1%
4.3.8. Our facility often hosts awareness campaigns about cervical cancer	29%	34%	11%	13%	13%
4.3.9. Our facility often participates in outreach awareness campaigns about cervical cancer	30%	40%	12%	6%	12%
4.3.10. I always encourage women to come for a Pap smear	54%	37%	6%	0	3%
4.3.11. Health management often provide training to improve my practice	21%	33%	20%	19%	7%
4.3.12. I feel competent in cervical screening practices	37%	45%	12%	5%	1%
4.3.13. Cervical screening programmes and/or practices are always monitored for standard compliance	31%	27%	19%	16%	7%

## Discussion

### Sources of information on the practice of cervical cancer screening

The workplace is a common source of information about CCS practices. The high response rate of ‘workplace’ as a major source of information is attributed to the element of workshops that are often conducted by Department of Health to offer PNs basic knowledge and skills on cervical cancer prevention and screening (South Africa 2017). These findings support those of a similar study conducted in Ethiopia, where 75.4% of nurses revealed that nurse training courses at the workplace were the primary source of information about CCS (Kieti 2016:44). Knowledge gained in the workplace is essential; however, such knowledge may not be comprehensive and is frequently not evaluated (Kokemuller 2017:4). A low number (4%) of PNs reported usage of mass media to obtain knowledge related to CCS; however, information found on media is not always accurate, filtered and evaluated (Gao & Hao 2017:2). Hence, the findings presented reluctance of the PNs to utilise such information. Formal and systematic training in cervical cancer control and prevention is required so that nurses can become more productive in the majority of areas of cervical cancer control and prevention. Makhubo and Naidoo (2020:5) recommended a thorough and endorsed training that focuses on reinforcement of institutional guidelines and protocols in CCS and prevention to ensure that healthcare workers (particularly PNs) are always resourceful in CCS and prevention.

### Knowledge of cervical screening practice

On CCS, nearly half of the respondents had good knowledge, some had very good knowledge and only a few had very poor knowledge. Respondents who were married and aged 40–44 years were found to have more knowledge about CCS than single young respondents. These findings are consistent with the findings of Mignote et al. (2015:1), who discovered that the majority of married nurses in public health institutions were well versed in CCS. Storr (2015:1) states that a person who is 40 years and above and married usually tends to develop more sense of responsibility; hence, they attain and sustain knowledge for both personal and professional development. Furthermore, when it came to knowledge about cervical cancer prevention, the majority of respondents were well versed. This study found that nurses are well trained in cervical cancer prevention; however, South Africa is still far behind in its goal of preventing cervical cancer in 70% – 80% of vulnerable women (Bogers et al. 2017:3). According to the country’s health policy, more than 70% of targeted women should be screened in order to successfully prevent cervical cancer (WHO 2015:3). As a result, these findings show a discrepancy between knowledge and actual practice of CCS and prevention in local clinics.

### Understanding of the Pap smear test for the detection of cervical cancer

The majority of respondents agreed that a Pap smear could be used to detect cervical cancer, with only 1% of the population unsure. Almost all nurses were aware of a Pap smear as a method of testing cervical cancer; comparable results were reported by Goyal et al. (2013:251), as 74% were aware of the Pap smear test, and 94% of nurses were also aware that cervical cancer was curable if detected early. Knowledge obtained from the workplace environment and nurse training courses may be attributed to the awareness of the curability of cervical cancer when detected early.

### Understanding of the risk factors for cervical cancer

The majority of respondents indicated that they were aware of cervical cancer risk factors such as sexually transmitted infections (95%), HIV (95%), multiple sexual partners (92%), human papilloma virus (HPV) (92%), early sexual debut (87%), many pregnancies and deliveries (71%) and low socio-economic status (58%). Most respondents were aware of the risk factors associated with cervical cancer; however, it was discovered that a significant number of respondents with less than 2 years of work experience were not fully aware that socio-economic status was also a major risk factor for cervical cancer. Combating cervical cancer on less literate, socio-economically poor and more vulnerable women requires reinforcement of primary and secondary preventive approaches, and PNs in PHC clinics are considered crucial drivers of these health programs (Tadesse 2015:2); therefore, inaccurate and unreliable information presented to communities may yield poor outcomes in the battle against cervical cancer. Low CCS promotion and uptake may be because of incomplete information provided by healthcare workers to women, as evidenced by some respondents’ lack of knowledge on risk factors such as low socio-economic status. According to Bruening et al. (2018:2), a health educator must provide relevant and accurate information to the targeted population in order to promote CCS.

### Understanding the frequency of Pap smears for HIV-negative women

According to the findings, the majority of respondents were aware of a 10-year interval for Pap smear tests for HIV-negative women; a small number of respondents responded with a 3-year or 1-year interval. The majority of respondents were aware that CCS in South Africa is done in decade intervals beginning at the age of 30. This result is consistent with the Cervical Cancer Prevention and Control Policy of South Africa (2017).

### Understanding the frequency of Pap smears for HIV-positive women

According to the findings, a large number of respondents were aware that Pap smears are performed at 3-year intervals for HIV-positive women; other responses were ‘1-year interval’ (45%). If the first Pap smear test was negative, HIV-positive women should be screened every 3 years (South Africa 2017:47). According to the findings, there is a thin line between responses of 3-year intervals and responses of 1-year intervals, with only a 4% difference. As a result, a significant number of respondents were unaware that, according to South Africa’s Cervical Cancer Prevention and Control Policy (2017), HIV-positive women should have a Pap smear every 3 years. This could imply that most HIV-positive women are receiving erroneous information about CCS; consequently, targeted women may be resistant to screening and will be less likely to recommend it to other community members, resulting in low uptake of CCS by the most vulnerable women. These findings have far-reaching implications for nurse practitioners. According to Khoza et al. (2016:5), if nurses are more knowledgeable and proactive in cervical cancer prevention efforts, they would be more likely to encourage women to use available screening services.

### Cervical cancer screening methods

Pap smear was specified as a method of CCS by all 83 (100%) respondents. These findings are supported by Olorunfemi (2017:10), who claims that the Pap smear is the oldest method of secondary prevention of cervical cancer that almost all health workers are aware of. The majority of nurses (75%) are aware of the availability of colposcopy as a screening method. Cervical biopsy was mentioned by nearly all nurses (81%) as an alternative to CCS. The high level of knowledge about available CCS methods may be attributed to extensive exposure to the workplace environment and in-service training.

### Cervical cancer screening management practices

In order to successfully manage CCS and prevention programs, the workforce and resources must be sufficient to meet counterpart screening demands. According to the findings of this study, the majority of respondents agreed that professional nurses are adequate for practice, while a few respondents strongly agreed that the personnel are adequate. The majority of respondents also strongly agreed that resources, particularly equipment and human resources, were regularly available. As a result of these findings, professional nurses and resources available at local clinics are adequate for CCS practice. The findings contradicted previous research that identified a shortage of nurses as one of the primary factors contributing to poor CCS implementation in PHC facilities (Akinyemiju et al. 2015:6; Chen et al. 2017:2; Munthali et al. 2015:505; Sibiya 2012:42). According to this study, a lack of nurses and resources may not be the sole cause of decline in CCS and prevention measures; therefore, other factors should be considered when evaluating CCS management practices.

In terms of carrying out CCS practices as part of their day-to-day duties, the majority strongly agreed, while a small number of respondents agreed. This finding is consistent with a previous study which found that nurses were expected to be early adopters of CCS and prevention practices (Grossniklaus et al. 2014:11549). When asked if they performed two or more Pap smears per shift, the majority of nurses disagreed, while others chose to be neutral. As a result, it was noted that the actual performance of Pap smears in the clinic did not occur frequently. This finding is supported by a similar study conducted in Nigeria, which discovered that nurses had good knowledge of CCS but practiced it infrequently (Anyebe et al. 2014:2). The health system of South Africa has been fragmented by various noncommunicable and communicable diseases, including HIV and AIDS. This creates a huge challenge in implementation of CCS, because PNs are obligated to treat and manage other diseases prevalent in the communities, resulting in a lesser amount of time dedicated to implementation of CCS (Garyfallos, Julien & Tafadzwa 2021:9).

This study backed up nurses’ claims that the majority of women who were screened for cervical cancer never returned for the results. Patients’ failure to follow up on results may be exacerbated by inaccuracy in information received from health workers. Coopoosmay et al. (2014:2) added that nurses should thoroughly explain cervical cancer risk factors, symptoms and complications to screened women, instilling the ‘vulnerability to cervical cancer’ perception so that they value the rationale for returning for results.

In terms of CCS promotion, the majority of respondents stated that their PHC facilities frequently host cervical cancer awareness campaigns and also participate in outreach campaigns. According to Owoeye and Ibrahim (2013:8), there should be a direct proportional relationship between CCS awareness and practice, which means that the more people who are aware of the benefits of CCS, the more people who seek CCS in local clinics (Chabanku et al. 2018:4; Vhuromu 2017:114).

The majority of respondents acknowledged management’s assistance in keeping them informed and competent about CCS and practices. A large number of nurses had learned about CCS and practised it in their workplace. This could refer to workshop, in-service training, briefing, mentorship and other services provided by nurse managers. Previous studies have found nurses to be well informed about CCS, which supports these findings (Anyebe et al. 2014:35; Grossniklaus et al. 2014:11549; Mignote et al. 2015:1).

Finally, the majority of respondents strongly agreed that CCS practices are frequently monitored and evaluated for standard compliance; the remaining 19% of the study population was neutral on this question. These findings demonstrate convincingly that the majority of nurses stated that CCS practices are monitored for standard compliance. According to the logical model framework (LFA) used in this study, when an effort is made to improve the functioning of the health program, positive results are expected (Finley 2015:5). To date, South Africa and the majority of developing countries are unable to screen for cervical cancer in 70% (or more) of women who are at risk (Bogers et al. 2017:3; Khoza et al. 2016:6). Furthermore, in most cases, CCS programs lack quality assurance structures to monitor screening practices (HPV Information Center 2018:60). Aside from nurses’ acknowledgement of practice monitoring in their clinics, the course’s productivity appears hazy. More research is needed to establish a link between CCS programs’ monitoring and evaluation and actual practice.

## Limitations

The sample was drawn from professional nurses who were on duty, and measurement errors may have occurred as a result of random interruptions from their work-related activities. Because of boundary and budgetary constraints, the study was limited to urban and semirural PHC clinics, and thus the findings cannot be generalised elsewhere.

## Recommendations

### Practice of nursing

The following recommendations were made based on the study’s findings:

- Nurses should educate their coworkers, families, communities, schools and children about cervical cancer and the importance of screening.
- Professional nurses and clinics should collaborate with community leaders (e.g. pastors, activists, politicians, etc.) to advocate for use of CCS so that women will be inspired and encouraged to participate in screening.
- To promote the use of CCS services, PHC facilities must collaborate with nonprofit organisations, as they play a crucial role in the extended efficient practice.
- Primary healthcare facilities must have a room specifically designed for Pap smear tests to ensure the privacy and comfort of the women being screened.
- Nurse managers must delegate duties on a daily basis, with a specific number of nurses only assigned to CCS services to enhance efficient care.
- Professional nurses should use the digital platforms from their smartphones and computers (e.g. watch skill videos on YouTube) to hone their Pap smear skills by watching demonstrated videos of skill performance.
- Nurse managers should monitor and evaluate CCS service implementation on a regular basis and report to the uMgungundlovu health district to ascertain strengths and shortcomings of the practice.

### Education in nursing

The following recommendations were made based on the study’s findings:

- Nursing regulatory bodies and the Department of Health should include a formal module on cervical cancer and screening in the nursing basic course curriculum.
- Professional nurses must express their views on Pap smear training concepts included in the nursing basic course curriculum.

The study uncovered and measured educational needs required by nurses. This study intended to translate research findings into evidence-based educational practices, thereby improving the faculty of nursing education, as well as nursing practice (AbuAlrub et al. 2021:2)

### Department of Health

The following recommendations were made based on the study’s findings:

- The Department of Health should strengthen periodic monitoring and evaluation on PNs who have been exposed to CCS skills training to improve screening practice.
- The government should increase the number of mobile clinics that offer CCS services.
- The Department of Health should create and advertise positions for professional nurses who will be dedicated solely to providing CCS services.
- The government should collaborate with nongovernmental organisations (NGOs) to educate the public and encourage the use of CCS services. To ensure that operational resources are well sustained, the Department of Health should increase budget allocation for facilities providing CCS services.
- The Department of Health should assess the use of a supermarket approach in clinics and applaud the efficient approach that will aid in the smooth implementation of CCS services.

## Conclusion

Respondents were knowledgeable about CCS; however, there was a knowledge gap among respondents regarding risk factors and recommended screening frequencies for cervical cancer. Staff and other operational resources were found to be adequate for CCS practice, despite previous studies identifying staff and resource shortages as a major barrier to CCS practice implementation. Despite having a good understanding of cervical cancer and screening, the actual practice of CCS was found to be low. This indicates that there are gaps between respondents’ knowledge and actual CCS practice. In this case, the researcher has suggested that qualitative studies be conducted to investigate potential barriers to CCS practice faced by nurses in their PHC facilities.
